# Gene expression profiling, prognosis, and immune microenvironment of KLF4 in malignancies

**DOI:** 10.1371/journal.pone.0322523

**Published:** 2025-04-29

**Authors:** Shoukai Yu, Lingmei Qian, Tianlang Tong, Xujun Ma

**Affiliations:** 1 Hongqiao International Institute of Medicine, Shanghai Tongren Hospital and Clinical Research Institute, Shanghai Jiao Tong University School of Medicine, Shanghai, China; 2 Harvard T.H. Chan School of Public Health, Harvard University, Boston, Massachusetts, United States of America; The First Affiliated Hospital of Nanjing Medical University, CHINA

## Abstract

Gene expression profiling plays a crucial role in understanding the role of Krüppel-like factor 4 (KLF4) in the prognosis and tumor immune microenvironment of various malignancies. The transcription factor KLF4 plays a crucial role in various cellular processes, including cell differentiation, proliferation, and apoptosis. Genetic alterations and aberrant KLF4 expression have been observed in many malignancies, thus suggesting a potential role as a prognostic marker and therapeutic target. Herein, a systematic analysis of KLF4 genetic alterations revealed the mutation, amplification, and deletion frequencies across different cancer types. The genetic alteration patterns varied across malignancies, thus highlighting the diverse roles of KLF4 in different tumor contexts. Secondly, the prognostic significance of KLF4 expression was assessed in multiple cancers. High expression levels of KLF4 were associated with better clinical outcomes in kidney renal clear cell carcinoma, while low KLF4 expression correlated with a favorable prognosis in certain malignancies. In conclusion, the genetic alterations, dysregulated RNA expression, and prognostic implications of KLF4 in malignancies underscore its significance in cancer biology. The present findings will aid in understanding the role of KLF4 in tumor biology and its association with immune responses. Future investigations should focus on elucidating the functional roles and regulatory mechanisms of KLF4 to further assess its potential as a therapeutic target and predictive biomarker in cancer management.

## Introduction

Kruppel Like Factor 4 (KLF4) is a zinc finger protein that regulates diverse cellular processes and plays a key role in the induction of macrophage differentiation [1]. KLF4 is also important in vascular health and aging, and its expression declines with age in the vascular endothelium. Furthermore, aberrant KLF4 expression has been associated with various cancers, including cancer stem cells [2].

KLF4, a zinc fingered transcription factor, has a crucial role in regulating cell proliferation, differentiation, and stem cell self-renewal. It is part of a group of four factors (Klf4, Oct4, Sox2, and c-Myc) that can stimulate the production of pluripotent stem cells [2]. KLF4 also contributes to cell fate reprogramming and stem cell self-renewal and is considered essential to maintaining cancer stem cells [4]. KLF4 has been shown to inhibit cell growth in various cancers [3]. Interestingly, in many tumors, KLF4 expression is often reduced or lost, which is associated with poor tumor differentiation. Moreover, this reduction in KLF4 expression in cancer cells is believed to be caused by genetic and epigenetic alterations [4]. While KLF4 has been implicated in many cancers, the significance of alterations in KLF4 expression across a broad range of tumors requires further examination.

In this study, a pan-cancer analysis was conducted to investigate the potential impact of KLF4 aberration in human malignant tumors. To the best of our knowledge, this is the first pan-cancer analysis of KLF4 across different cancers. Herein, several databases and bioinformatics analyses were performed to elucidate alterations in KLF4 expression, variant diversities, and clinical significance. Moreover, this study examined gene alterations and mutations, protein expression, survival prognosis, gene pathway enrichment, and immune infiltration. We believe that the findings presented herein will further clarify the potential role of KLF4 as a prognostic marker and its molecular mechanism in human cancers.

## Methods

### Examination of differential KLF4 expression

Initially, to investigate differential KLF4 mRNA expression between tumor and normal tissue, RNA-sequencing (RNA-seq) datasets derived from The Cancer Genome Atlas (TCGA) project were evaluated using the tumor immune estimation resource version 2 (TIMER2, http://timer.cistrome.org/). For TCGA datasets with no or very limited normal samples, corresponding normal tissues from the Genotype-Tissue Expression (GTEx) database were utilized and TCGA and GTEx datasets were evaluated using Gene Expression Profiling Interactive Analysis 2.0 (GEPIA2) [5]. GEPIA2 was also used to investigate KLF4 expression at different pathological stages across the examined cancer types using the “Pathological Stage Plot” module (http://gepia2.cancer-pku.cn/).

To further investigate potential associations between tumor and normal tissues, KLF4 total protein expression datasets obtained from the Clinical Proteomic Tumor Analysis Consortium (CPTAC) were examined with UALCAN [5]. UALCAN is an interactive web resource that can be used to explore cancer OMICS data, including TCGA and CPTAC datasets. The cancer datasets that contained KLF4 expression data included breast cancer, uterine corpus endometrial carcinoma (UCEC), pancreatic cancer, head and neck squamous carcinoma, and lung adenocarcinomas. To minimize potential batch effects and platform biases when integrating TCGA and GTEx datasets, we applied batch effect correction using the ComBat method and standardized the data through Z-score normalization. Quality control checks were also performed to ensure data consistency across the two platforms before proceeding with the analysis.

### Survival prognosis of KLF4

Survival analyses were conducted using GEPIA2 to generate overall survival (OS) and disease-specific survival (DSS) plots using TCGA datasets, with the expression threshold cutoff set to 50% to separate high- and low-expression cohorts. Log-rank tests were applied for hypothesis testing. Within the “survival analysis” module in GEPIA2, OS and DSS associated *p*-values, *q*-values, and Kaplan–Meier plots were acquired. All statistical analyses were performed using the “survival” package in R software.

### DNA methylation and genetic alterations analyses

To evaluate KLF4 epigenetic regulation, DNA methylation levels were investigated using UALCAN and based on obtained TCGA datasets with two transcription start sites (TSS200 and TSS1500), with Illumina IDs cg03267342 and cg13894301 [6]. Further genetic alterations, including mutations and copy number alterations (CNAs), were examined using the cBio Cancer Genomics Portal (http://cbioportal.org). The results include the mutations, amplifications, profound deletions of KLF4 and three-dimensional (3D) structure of KLF4 mutations.

### Immune infiltration analysis of KLF4

The “Immune-Gene” module within the TIMER2 web server was employed to investigate potential associations between KLF4 expression and immune cells, CD8 + T cells, and cancer-associated fibroblast infiltration. Immune infiltration estimates were determined using ten algorithms (TIMER, EPIC, MCP-COUNTER, CIBERSORT, CIBERSORT-ABS, QUANTISEQ, and four XCELL related algorithms), with purity-adjusted Spearman’s rank analysis performed and associated *p*-values and correlation values determined.

### Immune microenvironment analysis

Pearson’s correlation between genes and immunoinfiltration scores were calculated using the corr.test function in the R package_psych, with the immunoinfiltration scores derived from 10,180 tumor samples from 44 tumor types. The corresponding clinical features were also downloaded from the University of California Santa Cruz (UCSC, https://xenabrowser.net/) database. Next, the correlation expression data for the ENSG00000136826 (KLF4) gene and 44 marker genes belonging to three classes of RNA modification (m1A, m5C, m6A) were extracted. The sample sources were as follows: primary solid tumor, primary tumor, primary blood derived cancer-bone marrow, and primary peripheral blood-derived cancer All normal samples were filtered and log2(x + 0.001) transformed. Next, we calculated the Pearson correlation between ENSG00000136826 (KLF4) and five classes of immune pathways.

The Sangerbox platform (http://sangerbox.com/) was employed to evaluate any significant associations between KLF4 expression and the infiltration of 28 distinct immune cell subtypes. The ESTIMATE algorithm [7] was employed to evaluate immune cell infiltration and tumor purity. This algorithm utilizes the specific signatures that are associated with immune and stromal infiltration and is based on single sample gene set enrichment analysis (ssGSEA). These scores then form the basis for the ESTIMATE score, which reflects tumor purity. Expression for the KLF4 gene and 60 genes belonging to two types of immune checkpoint pathways, to include associated markers, were extracted. The strength of the correlation is represented by color, while asterisks denote statistically significant p-values obtained through Spearman correlation analysis (*P < 0.05; **P < 0.01; ***P < 0.001).

The DNA methylation-based stemness score (DNAss) was calculated based on tumor methylation characteristics and based on TCGA datasets. We integrated the sample stemness index and gene expression data, and further transformed each expression value by log2(x + 0.001). Finally, tumor cohorts with less than three samples were eliminated. Additionally, DNAss, RNA stemness score (RNAss), and RNA-Seq data, along with clinical data, were downloaded from the UCSC Xena database. The DNAss, which represents the DNAss of cuproptosis-associated genes, was assessed using the R packages “limma” and “corrplot,” with Spearman’s analysis also employed. Tumor epigenetic features were evaluated based on the DNAss, while gene expression was evaluated based on the RNAss. The scores ranged from 0 to 1, with 0 indicating high differentiation and 1 indicating undifferentiated. Subsequently, the DNAss and RNAss were analyzed among the given tumor cohort subgroups.

### Gene-related enrichment analysis

The STRING database (https://string-db.org/) was employed to identify the 50 top potential KLF4-binding proteins for further analyses. Next, GEPIA2 was employed to identify the 100 top genes with an expression pattern similar to that of KLF4 in all TCGA tumor and normal samples. Pearson correlations were performed and associated *p*-values and correlation coefficients were determined.

A cnet plot was generated using TIMER2, to include *p*-values and partial correlations in the purity-adjusted Spearman’s test. A Venn diagram was employed to conduct an intersectional analysis of KLF4 binding and interacting genes base on STING and GEPIA2 findings. Gene Ontology (GO) and Kyoto Encyclopedia of Genes and Genomes (KEGG) enrichment analyses were performed using the “clusterProfiler” package in R statistical software version 4.0.5, and bubble plots were created using the “tidyr” and “ggplot2” packages.

## Results

### Gene expression analysis

**Fig 1:**
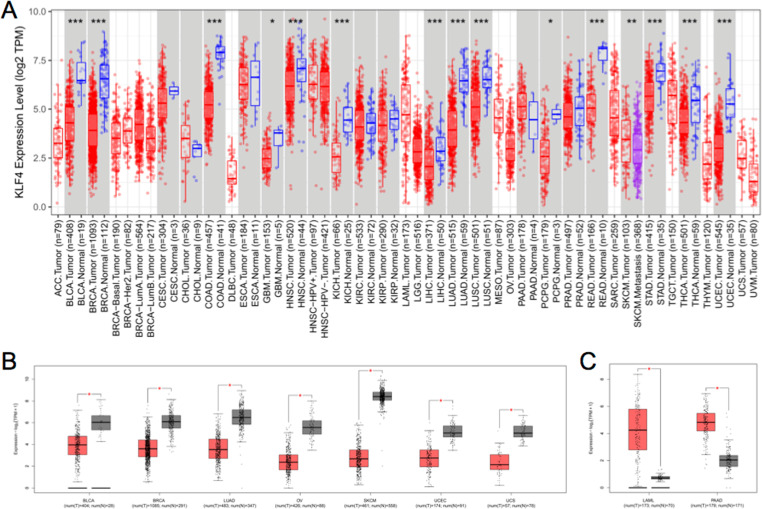
Analysis of KLF4 RNA expression in various tumors utilizing TCGA and GTEx datasets. (A) Assessment of KLF4 expression in different cancer types using TIMER2. Statistical significance is denoted as *p < 0.05, **p < 0.01, and ***p < 0.001. (B) and (C) TCGA and GTEx datasets were examined using GEPIA2, with corresponding normal tissues from the GTEx database utilized as control samples for TCGA cancers with little or no control samples. Statistical significance is denoted as *p < 0.001. In TCGA dataset, expression levels were significantly lower in the cancer groups relative to the normal tissues, while in the GTEx dataset, the opposite was noted.

Initially, KLF4 expression was examined across different cancer types using TCGA datasets with the TIMER2 server. The results showed that KLF4 expression is significantly downregulated in BLCA, BRCA, COAD, HNSC, KICH, LIHC, LUAD, LUSC, READ, STAD, THCA, and UCEC tumor tissues relative to their corresponding normal tissues (Fig 1A, *p* < 0.001). When combining TCGA and GTEx datasets to compensate for cancers with little to no normal samples, BLCA, BRCA, LUAD, OV, SKCM, UCEC, and UCS were identified as having significantly decreased KLF4 expression using GEPIA2 (Fig 1B, *p* < 0.001); while LAML and PAAD were significantly increased relative to their corresponding normal tissues (Fig 1C, *p* < 0.001).

The “Pathological Stage Plot” module of GEPIA2 was employed to determine whether KLF4 expression differs based on tumor pathological stage. The results demonstrated that KLF4 expression is significantly associated with clinical stage in six of the examined cancer types: BRCA (*p *= 0.0384), KIRC (*p* = 2.68e − 07), KIRP (*p* = 1.02e − 06), PAAD (*p* = 0.0126), TGCT (*p* = 0.0224), and THCA (*p* = 0.0393) (Fig S1 in S1 File). Furthermore, the results of the CPTAC dataset showed lower KLF4 total protein expression in the primary tissues of HNSC and LAUD (Fig S2 in S1 File, *p* < 0.05) relative to the normal controls, while higher KLF4 expression was noted in PAAD primary tissues (*p* < 0.05) relative to the normal controls.

### Analysis of survival prognosis

OS and DSS plots were generated to determine the prognostic potential of KLF4.

**Fig 2:**
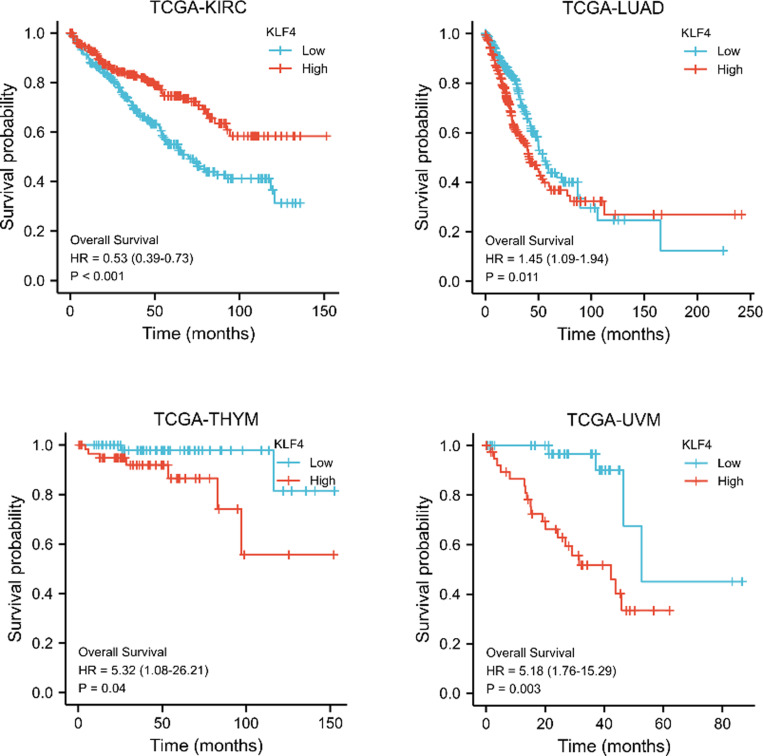
Survival outcome analysis in malignancies displaying abnormal KLF4 expression.

The results of OS showed that highly expressed KLF4 is linked with a poor overall prognosis in THYM (*p* = 0.04) and UVM (Fig 2, *p *= 0.003), while low KLF4 expression is significantly correlated with a poor prognosis in KIRC (*p* < 0.001). Interestingly, the results also showed that high KLF4 expression is significantly correlated with a poor prognosis in the early stages in LUAD (*p* = 0.011). Additionally, DSS analysis (Fig S3 in S1 File) showed that high KLF4 expression is significantly correlated with a poor prognosis in LUAD (*p* = 0.008) and UVM (*p* = 0.003), while low KLF4 expression is significantly correlated with a poor prognosis in KIRC (*p* < 0.001) and KIRP (*p* = 0.008). Both the OS and DSS plots revealed some direct associations between KLF4 expression levels and prognosis.

### DNA methylation and genetic alteration analysis

UALCAN was utilized to investigate potential methylation alterations at the KLF4 promoter (Fig S4 in S1 File). Interestingly, respiratory system-related tumors, such as LUAD and LUSC, exhibited an increase in DNA methylation. For the tumors with upregulated KLF4 (LAML and PAAD), the DNA methylation level could not be predicted based on the mRNA expression features. Additionally, due to the unavailability of a DNA methylation dataset for KICH and UVM normal controls, comparative analysis was conducted across different patient populations. These findings confirm that aberrant KLF4 expression is not solely attributed to DNA methylation. Thus, further investigations could be conducted to explore histone modifications [5] and glycosylation [6].

**Fig 3:**
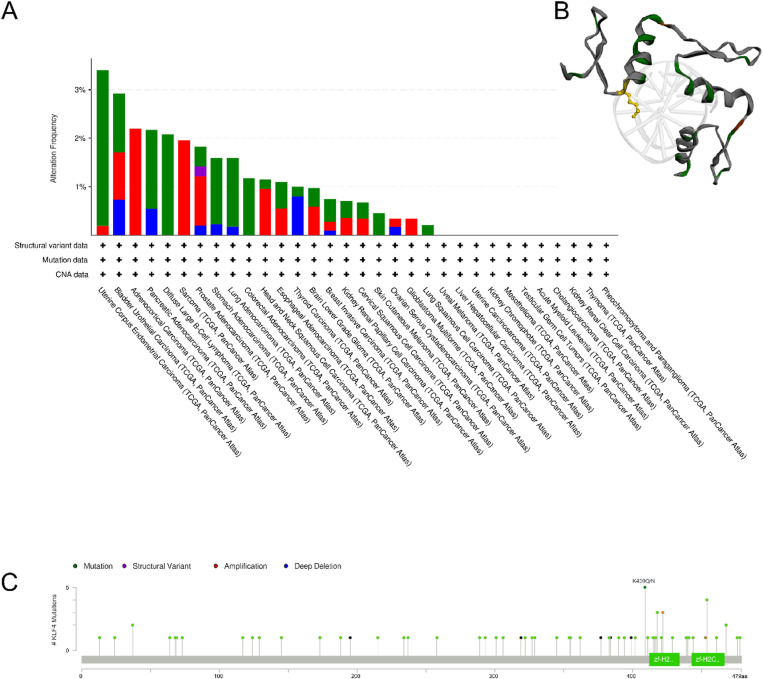
Analysis of KLF4 alterations using the cBio portal. (A) Analysis of genetic variations in KLF4 across different tumor types. (B) Visualization of the three-dimensional structure of KLF4 mutations, with the mutation R163*/Q highlighted in yellow. (C) Distribution of KLF4 mutations across all exons without a prominent hotspot mutation site being noted in TCGA cohort.

Genetic alterations in KLF4 were examined using the cBio portal, with 10,953 samples out of 10,967 samples from TCGA database for different cancers examined. The pan-cancer analysis of KLF4 revealed that the highest alteration frequencies are present in UCEC (3.4%) and BLCA (2.92%) (Fig 3A). The highest frequencies of mutations were predominantly found in UCEC, DLBC, and PAAD. Amplifications comprised the majority of alterations in ACC and SARC. Moreover, the highest frequencies of deep deletions were observed in BLCA and THCA (Fig 3A). The most frequently observed mutation was K409Q/N, and a 3D structure of this KLF4 mutation was constructed (Fig 3B). Further analysis revealed that the KLF4 mutations in various malignancies are distributed throughout without the presence of a mutational hotspot site (Fig 3C).

KLF4 alterations in each given tumor type occur at a very low frequency (Fig S5 in S1 File). Additionally, copy number variations were also found to not be significantly associated with KLF4 expression across all tumors (Fig S6 in S1 File). One possible explanation is that aberrant KLF4 expression is not directly influenced by genetic alterations.

### Analysis of total protein expression

CPTAC datasets were utilized for three types of tumors (HNSC, LUAD, and PAAD) to compare KLF4 total protein levels between normal and primary tumor tissues. The results showed significantly lower protein levels in primary tumor tissues compared to normal tissues for HNSC and LUAD (Fig S2 in S1 File, *p* < 0.05). Higher KLF4 expression was noted in the primary PAAD tissues (*p* < 0.05) relative to the normal controls.

### Immune infiltration analysis

Tumor infiltrating immune cells have been recognized as an important component of the tumor microenvironment and play a crucial role in tumor initiation, promotion, progression, and metastasis [8]. Cancer-associated fibroblasts have also been implicated in the modulation of various tumor infiltrating immune cells [10]. Thus, KLF4 expression and immune cell infiltration levels were examined across various TCGA tumor types using TIMER, CIBERSORT, CIBERSORT-ABS, QUANTISEQ, XCELL, MCPCOUNTER, and EPIC algorithms.

**Fig 4:**
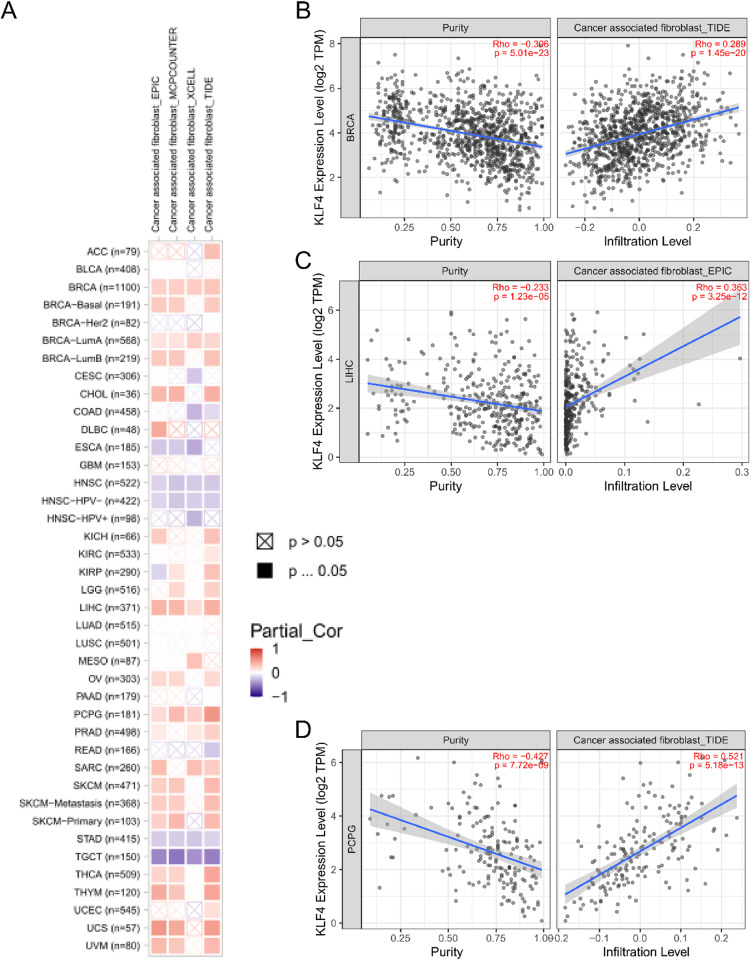
Evaluation of the relationship between KLF4 expression and cancer-associated fibroblast (CAF) infiltration. (A) Four computational algorithms (EPIC, MCPCOUNTER, XCELL, and TIDE) were employed to investigate the possible link between KLF4 expression and CAF infiltration in various cancer types. Positive correlations were then confirmed in (B) BRCA, (C) LIHC, and (D) PCPG via scatter plots.

The various analyses indicated that KLF4 expression is positively correlated with CD8 + T cell infiltration in HNSC-HPV- and UVM, while negatively correlated in LUSC and THYM, based on most of the algorithms (Fig S7 in S1 File). Additionally, KLF4 expression and cancer-associated fibroblast infiltration was found to be positively correlated in BRCA, BRCA-LumA, LIHC, and PCPG, while negatively correlated with HNSC, HNSC-HPV-, STAD and TGCT, based on all or most algorithms (Fig 4A). These findings were then confirmed for a subset of the tumors using scatter plots (Fig 4B–D). Moreover, KLF4 clinical significance was demonstrated in BLCA, BRCA, CESC, LAML, PAAD, PCPG, SKCM, THCA, UCEC, and UCS via ROC analysis (Fig S8 in S1 File).

### Immune microenvironment

The ESTIMATE algorithm was employed to evaluate the overall presence of immune cells. Our analysis revealed a notable decrease in ESTIMATE scores in metastatic tumors when compared to primary tumors (Fig S9 in S1 File), thus indicating a diminished immune cell infiltration in metastatic lesions. Furthermore, KLF4 expression was significantly correlated with immunoinfiltration in 32 cancer species, with six being significantly negatively correlated: TCGA-CESC (*N* = 291, *r* = −0.15, *p* = 9.6e − 3), TCGA-ESCA (*N* = 181, *r* = −0.20, *p* = 7.0e − 3), TCGA-STES (*N* = 569, *r* = −0.19, *p* = 3.3e − 6), TCGA-HNSC (*N* = 517, *r *= −0.20, *p *= 7.5e − 6), TCGA-LUSC (*N* = 491, *r *= −0.13, *p* = 3.6e − 3), TCGA-TGCT (*N* = 132, *r *= −0.34, *p* = 6.3e − 5).

RNA modifications can directly affect the chemical properties of the molecule, including its charge, base pairing, secondary structure, and protein-RNA interactions. These modifications can in turn regulate gene expression by controlling RNA processing, localization, translation, and eventual decay [9,10]. Herein, m6A (N6-methyladenosine), m1A (N1-methyladenosine), and m5C (5-methylcytosine) were the most common RNA methylation modifications. Pearson’s correlation analysis showed that m6A methylation plays a biological role mainly through RNA-binding proteins (Fig S10 in S1 File). Furthermore, when examining correlations between tumor KLF4 expression and immune checkpoint genes as a means to predict immunotherapy applications, a negative correlation between KLF4 and a wide range of immunomodulators was noted (Fig S11 in S1 File). Overall, the top four checkpoint genes associated with KLF4 and correlated with a tumor inhibitory effect in several tumors were C10orf54, CD274, IL10, and TGFB1. While the top three KLF4-assocaited genes correlated with a tumor stimulatory effect for several tumors were CX3CL1, TNFRSF4, and IL1A. Meanwhile, the top three genes that are least correlated with KLF4 and associated with a tumor inhibitory effect in several tumors were BTLA, IDO1, and LAG3. The KLF4-assocaited genes that were least correlated with a tumor stimulatory effect for several tumors included TNFRSF18.

Cells that possess stem-cell like characteristics, stemness, have a higher tendency toward tumorigenic behavior, to include DNA hypermethylation of specific promoters to suppress gene expression [11]. Thus, to discern potential correlations between KLF4 expression and DNAss, Pearson’s correlations were performed based on immune checkpoint genes and KLF4 expression using Sangerbox (*p* < 0.05). The results identified 14 significant associations, with MESO and PCPG being the most negatively correlated and THYM and TGCT being the most positively correlated (Fig S12 in S1 File). The correlation between KLF4 expression and cancer stemness scores (DNAss) based on the Pearson’s correlation analysis is shown in Fig S12 in S1 File, with only a few associations achieving statistical significance.

### Enrichment of KLF4-related partners

**Fig 5:**
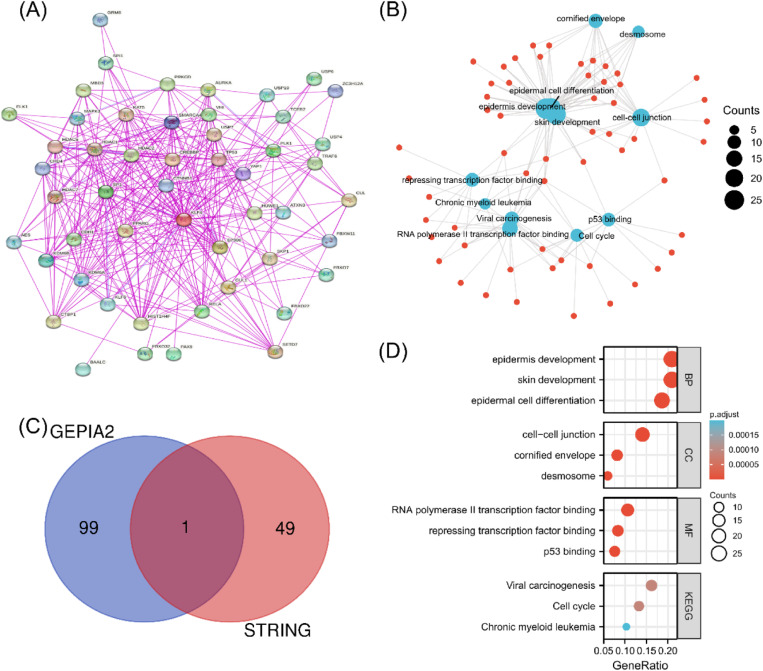
Enrichment analysis of the KLF4 gene. (A) Utilizing the STRING tool, 50 proteins were identified that interact with KLF4. (B) KEGG pathway analysis was performed based on the genes that interact with KLF4 identified from the STRING and GEPIUA2 analyses. (C) An intersection analysis was performed to identify genes that both bind to and are correlated with KLF4. (D) GO analysis was performed and the results were visualized by constructing a cnet plot.

To further explore the molecular mechanism of KLF4 in tumorigenesis, KLF4 binding and potential correlated expression was examined using several pathway enrichment analyses. The STRING tool identified a total of 50 experimentally supported binding proteins and an interaction network was constructed (Fig 5). To further expand the list, GEPIA2 was employed and the top 100 genes associated with KLF4 expression were determined using all TCGA tumor expression data.

These STRING and GEPIA2 datasets were then combined and further enrichment analysis was performed using KEGG and GO enrichment analyses. The KEGG analysis identified “Cell cycle” pathways as being associated with KLF4 (Fig 5B), while GO analysis identified biological processes such as “epidermis-development,” “skin-development,” and “epidermal-cell-differentiation” (Fig 5D).

Furthermore, an intersection analysis of the STING and GEPIA2 datasets identified one common member (Fig 5C). Notably, the KEGG pathways encompassed “cell cycle,” while the GO analysis identified “epidermis development,” “skin development,” and “epidermal cell differentiation” in association with the biological processes category. Based on existing literature, KLF4 has been shown to both promote and inhibit various stages of the cell cycle depending on the specific context and cell type. Experimental data suggest that KLF4 may regulate the G1/S transition by modulating cyclin expression and interacting with key cell cycle regulators, such as p21, suggesting a complex role in tumor progression.

## Discussion

Previous studies have demonstrated that KLF4 exhibits a cell type-dependent role in either tumor suppression or promotion [12]. Additionally, KLF4 has been identified as a prognostic predictor for urothelial carcinoma of the bladder, where it regulates TWIST1-mediated epithelial-mesenchymal transition [12]. However, the precise role of the multifunctional KLF4 in the molecular pathogenesis of various tumors remains unclear. Therefore, in this study, we employed a pan-cancer approach to identify alterations in KLF4 DNA sequence, gene and protein expression, and DNA methylation in more than 30 tumors. Notably, a significant downregulation of KLF4 expression was observed in BLCA, BRCA, LUAD, OV, SKCM, UCEC, and UCS. To the best of our knowledge, this is the first pan-cancer study to characterize KLF4 genetic and epigenetic variations in various human malignant tumors.

The CPTAC database was employed to identify differential KLF4 total protein expression between various primary and tumor samples (Fig S2 in S1 File). KLF4 was upregulated in PAAD relative to the normal control, while it was significantly downregulated in HNSC and LUAD. Furthermore, DSS analysis revealed that low KLF4 expression is associated with a poor prognosis for KIRP and KIRC. However, in LUAD and UVM, high levels of KLF4 expression were associated with an unfavorable prognosis.

Overall, this comprehensive investigation of KLF4 across multiple cancer types reveals its substantial clinical significance in terms of prognosis, protein expression, and immune cell infiltration in some cancers. Moreover, KLF4 appears to exert a crucial role in both cancer cell stemness and tumor development. Since KLF4 has been implicated as a potential immune-related molecule, it might serve as a potential prognostic marker. These findings indicate that further examination into the role of KLF4 in tumorigenesis is warranted to gain a deeper understanding into its role in the tumor microenvironment. The role of KLF4 in cancer is complex and context-dependent. In certain cancers, such as KIRC and LUAD, KLF4 expression is associated with opposing prognostic outcomes. High expression of KLF4 correlates with poor prognosis in KIRC, while in LUAD, higher expression may suggest a better outcome. These contradictory findings suggest that KLF4 may exhibit distinct roles depending on tissue-specific gene regulation, the tumor microenvironment, and interactions with other signaling pathways.

Future studies should explore the molecular mechanisms underlying these contrasting effects of KLF4, particularly focusing on how its function may switch between tumor suppressor and oncogene depending on factors such as the activation of the PI3K/AKT, Wnt/β-catenin, or TGF-β pathways. Understanding this dual role could provide valuable insights into the precise role of KLF4 in cancer progression and its potential as a therapeutic target. Regarding the negative correlation between KLF4 and immune checkpoint genes (such as CD274), while our analysis indicates this association, we have not yet conducted direct experimental validation. To further validate its predictive value in immunotherapy, we suggest incorporating this finding into public immunotherapy datasets (such as IMvigor210 cohort) for further cohort analysis. By leveraging these existing immunotherapy cohort data, we can more effectively assess the relationship between KLF4 and immune checkpoint genes, and explore its potential as a predictor of immunotherapy response.

In a previous study examining colorectal cancer, downregulated KLF4 expression was associated with a poor prognosis [13]. Herein, a similar association was observed in COAD and READ, but the association did not achieve statistical significance following OS/DSS analysis when combining the cohorts (Fig S12 in S1 File). Furthermore, while immune checkpoint upregulation is commonly linked to a poor prognosis, these tumors are more likely to demonstrate a favorable response to immune checkpoint inhibitors. The findings indicated that KLF4 should be considered a target of interest. Thus further characterizing the role of KLF4 will enhance our comprehension of the regulatory pathways governing immune checkpoint modulation and potentially enhance the success rate and effectiveness of immune checkpoint therapy. Furthermore, this study reinforces the results of previous meta-analyses [14], which found that KLF4 expression is not directly associated with the prognosis of all types of tumors. In other words, KLF4 operates through different mechanisms in different tumor types.

To further examine the mechanisms of KLF4, immune checkpoint genes were examined in association with KLF4 expression. Herein, several KLF4-assocaited genes, C10orf54 and CD274 were correlated with tumor suppression, while others, including CX3CL1, TNFRSF4, and IL1A, were correlated with tumor stimulation. In previous studies, BTLA, LAG3, and IDO1 have been identified as immune checkpoint inhibitors [15–18]. KLF4 plays an important role in tumorigenesis via immune regulation and promotes the cytolytic effector function in a subset of CD8 T cells [15]. KLF4 has also been shown to function in the re-activation of CD8 T cells in the immune response against cancer [16]. Collectively, the results suggest the higher expression of KLF4 indicates the positive correlation with cellular markers of immune activation. These results highlight the potential value of KLF4 as a clinical biomarker and a specific therapeutic target. The results of the current study show that KLF4 is associated with cancer prognosis and immune infiltration across several types of cancers, which is consistent with the findings of recent studies [17–19]. KLF4 expression is also strongly associated with immune-related gene expression in various cancers and may play a crucial role as a prognostic biomarker for different cancers and possible therapeutic target across several cancer types.

## Supporting information

S1 File The supporting information includes 12 supplementary figures detailing KLF4 expression, protein levels, survival outcomes, mutations, DNA methylation, immune interactions, and correlations with various molecular features across different cancer types. (DOCX)

## Abbrevations

ACC: Adrenocortical carcinoma
BLCA: Bladder Urothelial Carcinoma
BRCA: Breast invasive carcinoma
CESC: Cervical squamous cell carcinoma and endocervical adenocarcinoma
CHOL: Cholangiocarcinoma
COAD: Colon adenocarcinoma
DLBC: Lymphoid Neoplasm Diffuse Large B-cell Lymphoma
ESCA: Esophageal carcinoma
GBM: Glioblastoma multiforme
HNSC: Head and Neck squamous cell carcinoma
KICH: Kidney Chromophobe
KIRC: Kidney renal clear cell carcinoma
KIRP: Kidney renal papillary cell carcinoma
LAML: Acute Myeloid Leukemia
LGG: Brain Lower Grade Glioma
LIHC: Liver hepatocellular carcinoma
LUAD: Lung adenocarcinoma
LUSC: Lung squamous cell carcinoma
MESO: Mesothelioma
OV: Ovarian serous cystadenocarcinoma
PAAD: Pancreatic adenocarcinoma
PCPG: Pheochromocytoma and Paraganglioma
PRAD: Prostate adenocarcinoma
READ: Rectum adenocarcinoma
SARC: Sarcoma
SKCM: Skin Cutaneous Melanoma
STAD: Stomach adenocarcinoma
STES: Stomach and Esophageal carcinoma
TGCT: Testicular Germ Cell Tumors
THCA: Thyroid carcinoma
THYM: Thymoma
UCEC: Uterine Corpus Endometrial Carcinoma
UCS: Uterine Carcinosarcoma
UVM: Uveal Melanoma.
