# Supplementary material for: Gene expression profiling, prognosis, and immune microenvironment of KLF4 in malignancies
Source: PLoS One. 2025 Apr 29;20(4):e0322523. doi: 10.1371/journal.pone.0322523 (PMC12040095; doi:10.1371/journal.pone.0322523)
Supplement: S1 File — (DOCX) [file pone.0322523.s001.docx]

**Supplementary Figures**

**
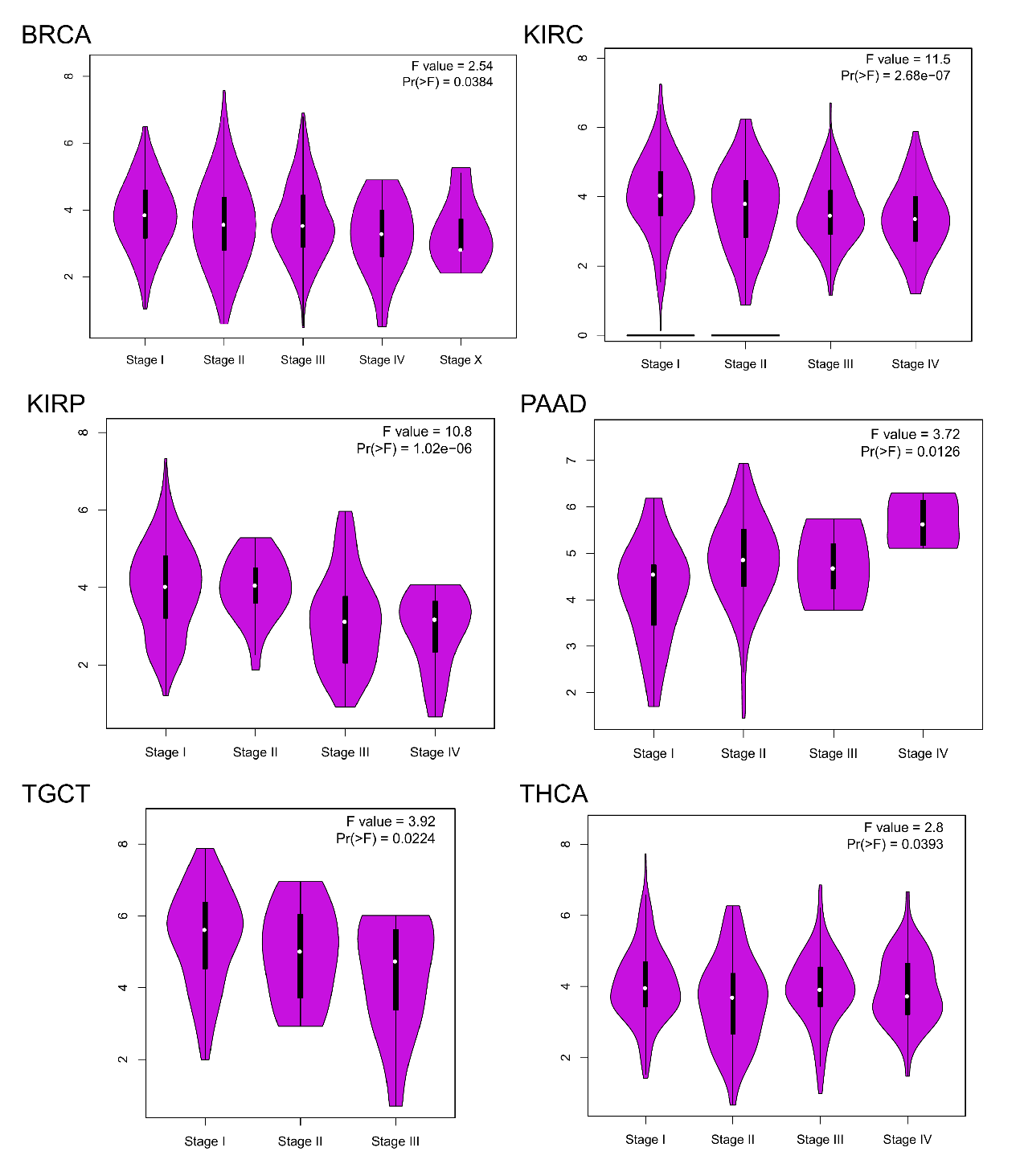
**

**Figure S1** *KLF4* expression was analyzed base on pathological stage in BRCA, KIRC, KIRP, PAAD, TGCT, and THCA using TCGA datasets. A Log2 (TPM +1) transformation was applied.


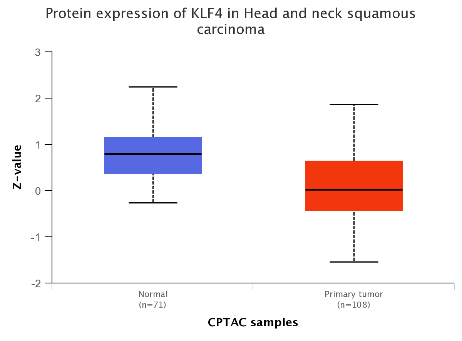

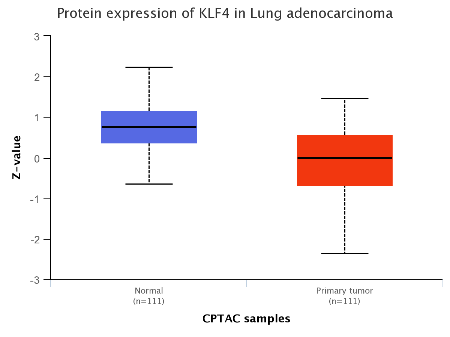

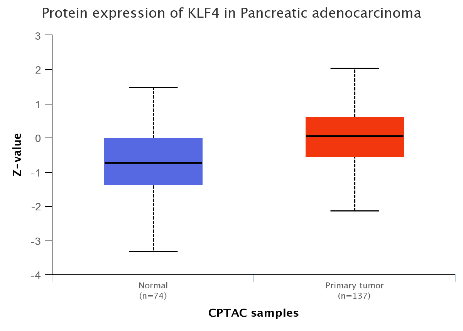

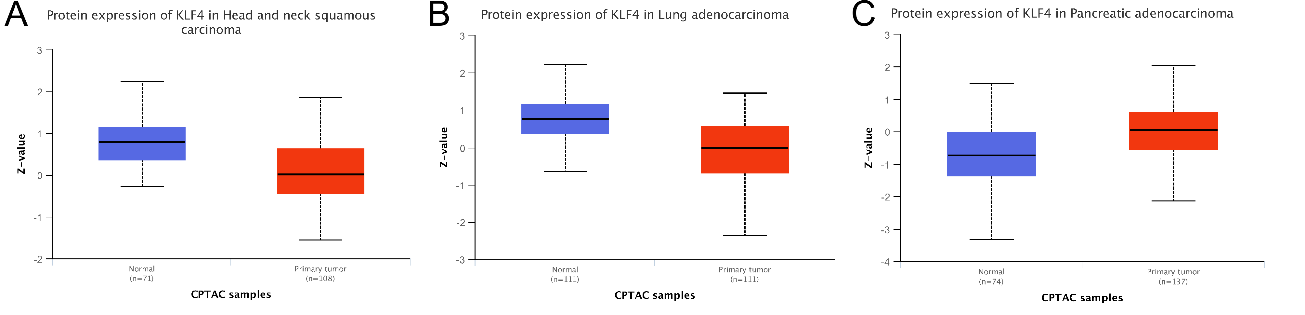


**Figure S2** Examination of KLF4 total protein expression between normal and primary tumor tissues from different cancer types and based on the CPTAC dataset (*p* < 0.05).


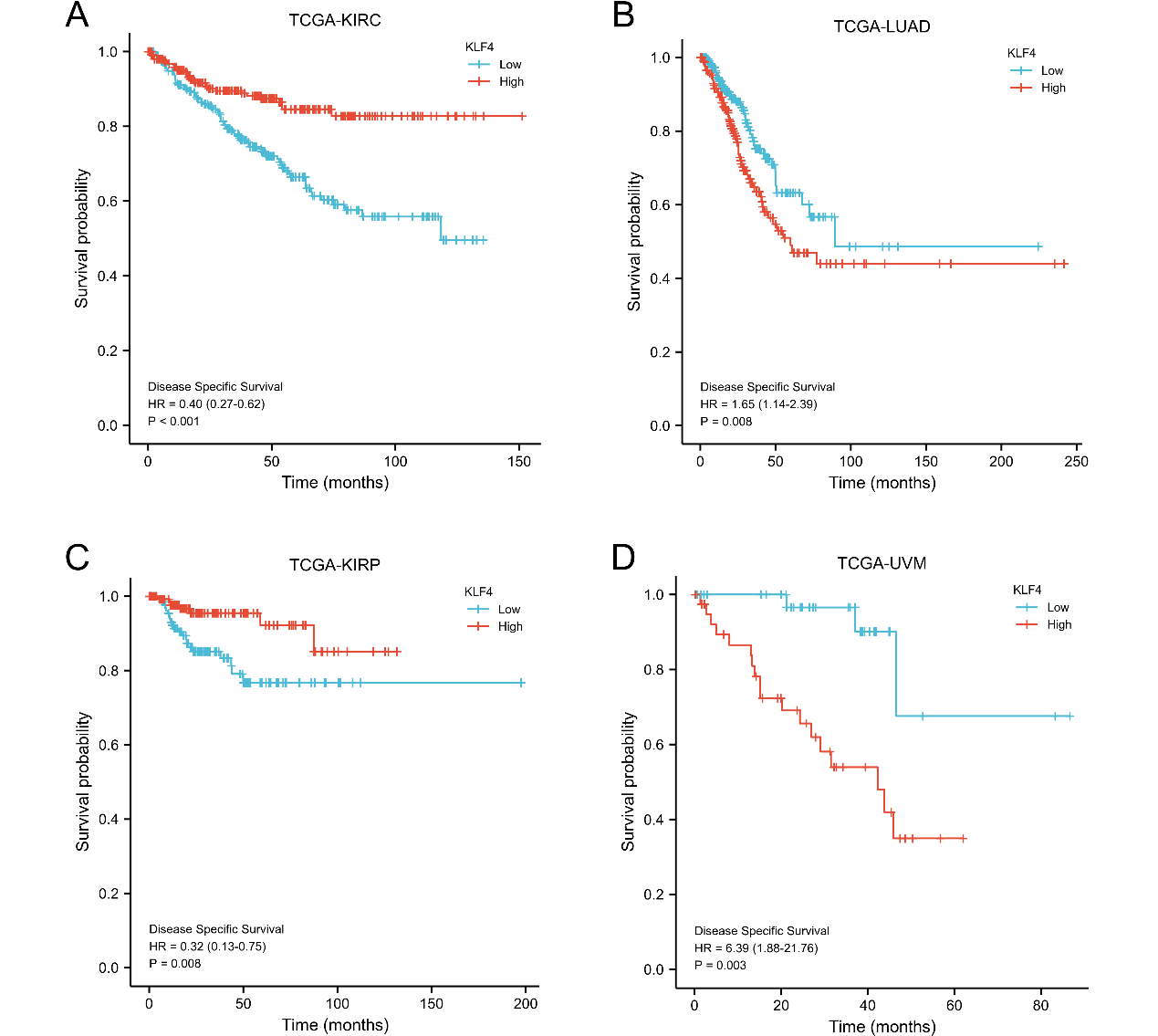


**Figure S3** Disease-specific survival (DSS) data for KLF4 in different tumors.

**.**


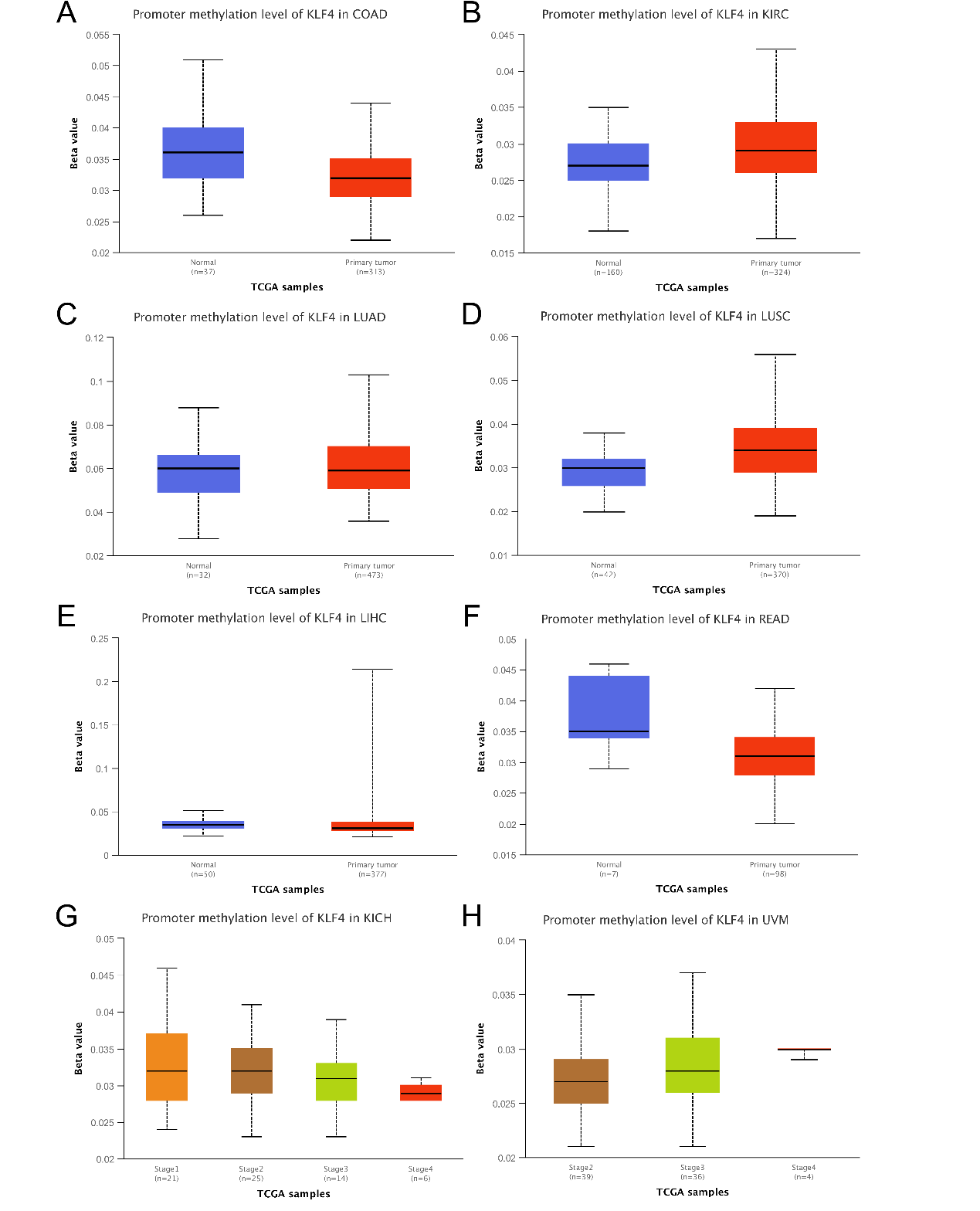


**Figure S4** Analysis of KLF4 DNA methylation levels in different tumors.


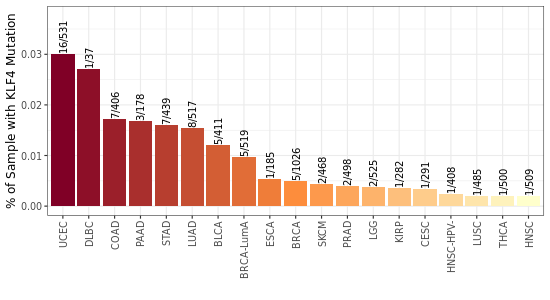


**Figure S5** KLF4 mutational frequencies in different cancer types.


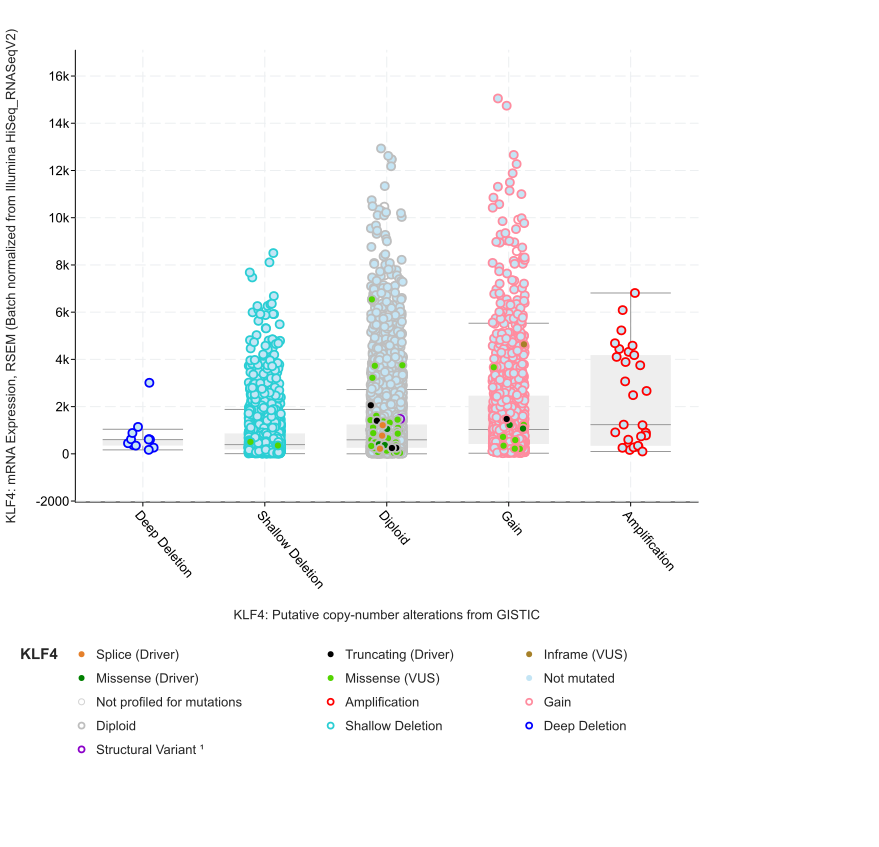


**Figure S6** Lack of statistical relevance between DNA copy variations and RNA expression of KLF4 in most cases.


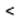

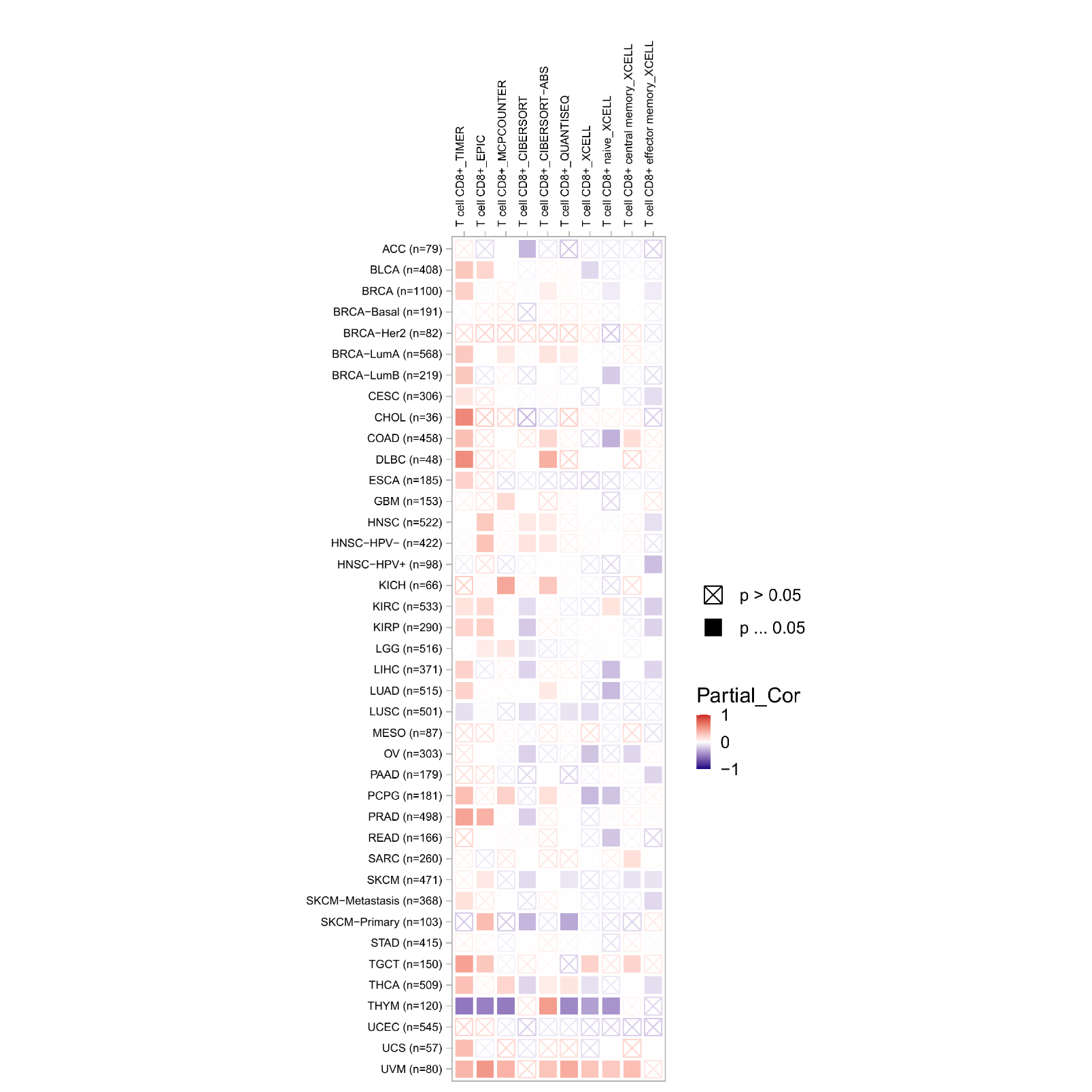


**Figure S7** Investigation of the relationship between KLF4 expression and immune infiltration of CD8+ T cells based on ten algorithms across various cancer types using TIMER2.


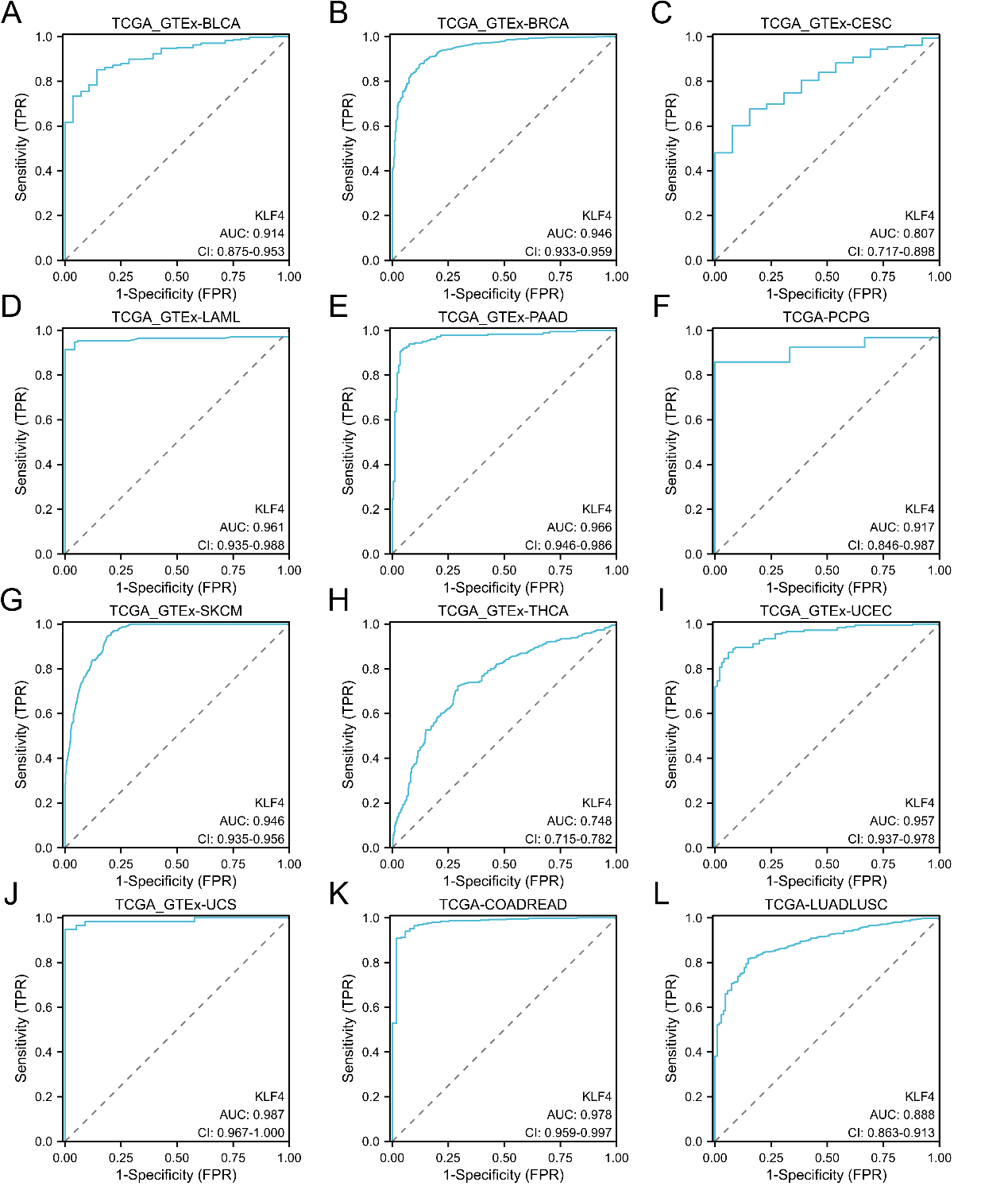
**Figure S8** ROC analysis to examine the prognostic ability of KLF4 in several tumor types.


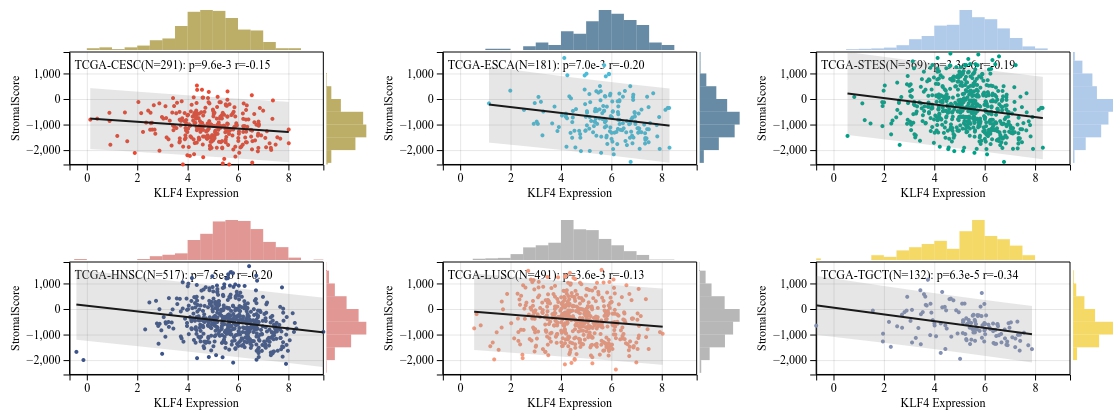


**Figure S9** Correlations between the ESTIMATE scores and KLF4 expression.


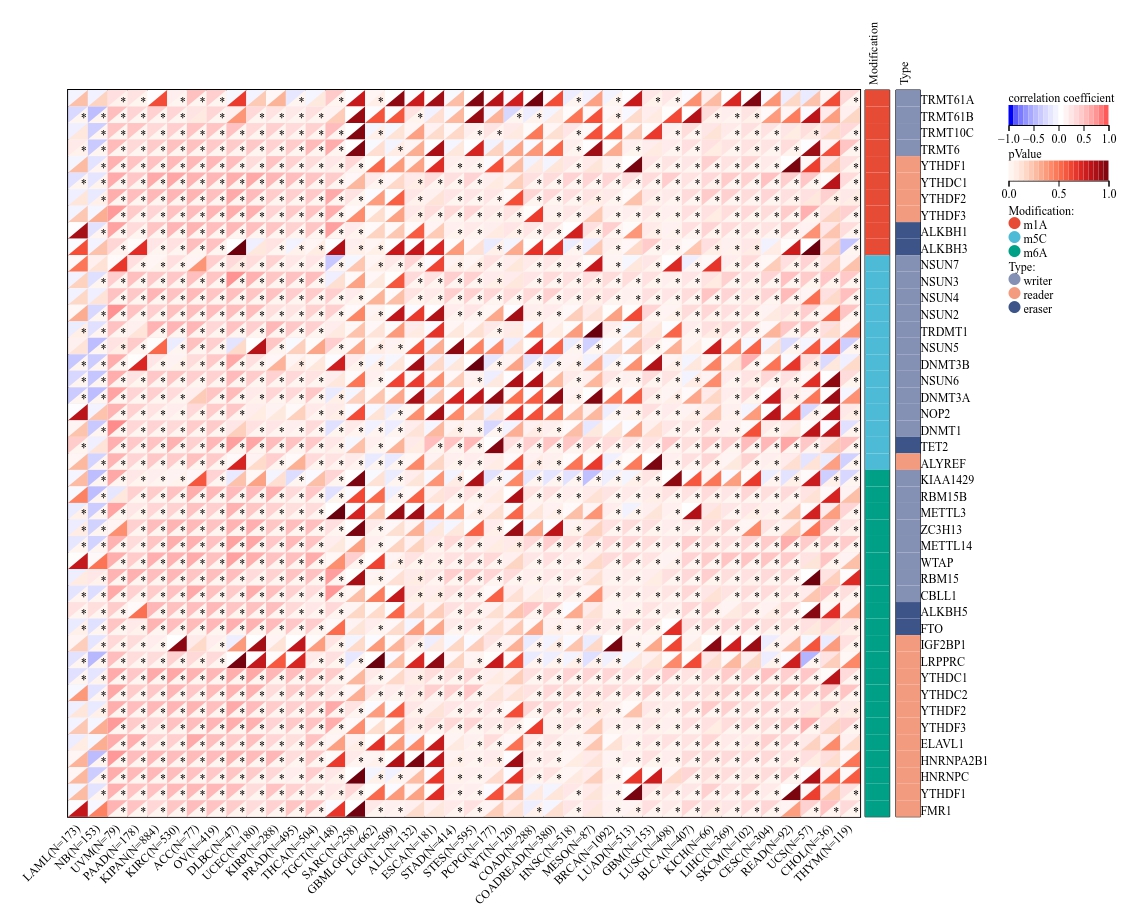


**Figure S10** Correlation analysis of RNA modification genes associated with m6A, m5C, and m1A and KLF4 expression using Sangerbox. **p* < 0.05.


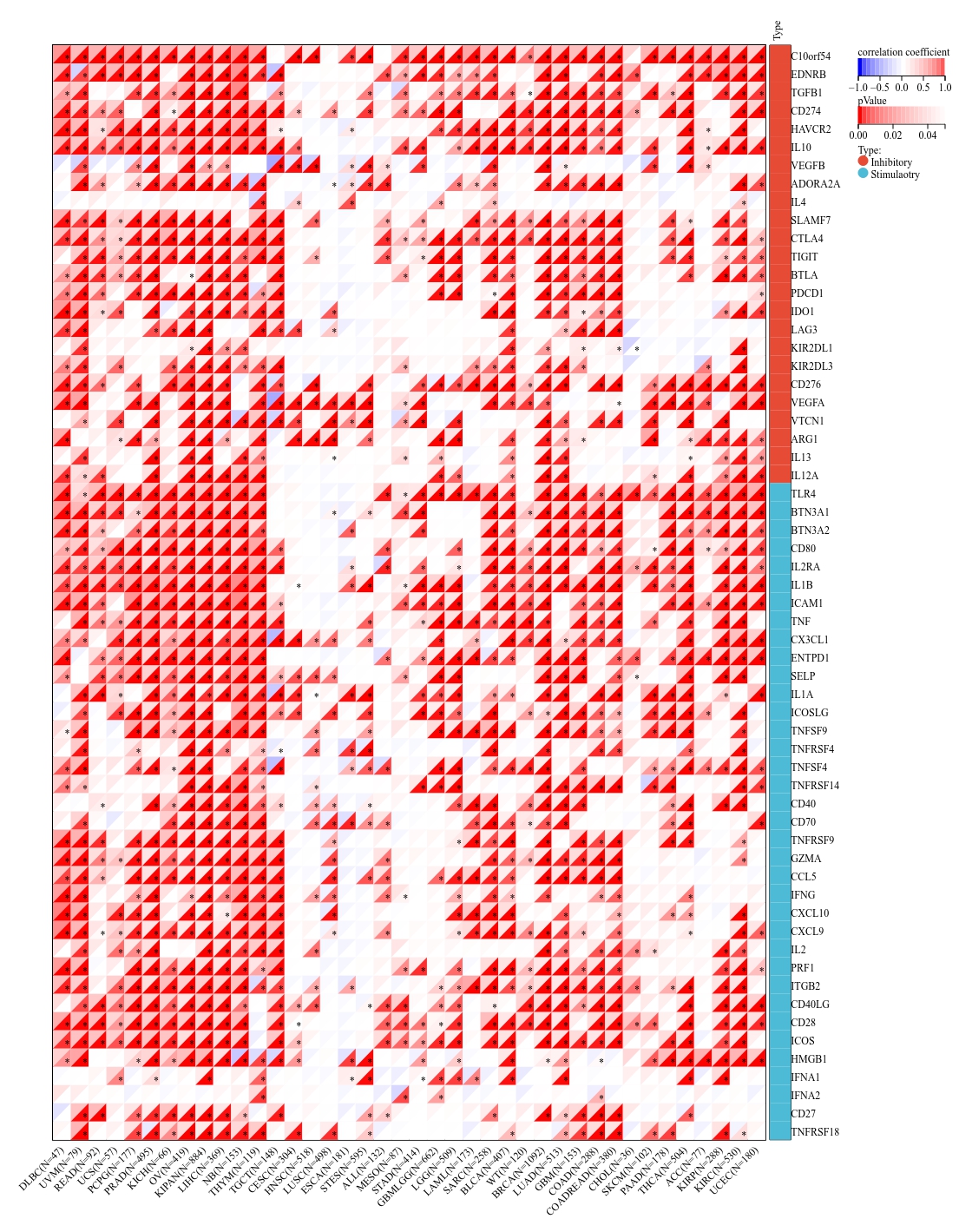


**Figure S11** Correlation analysis of immune checkpoint genes and KLF4 expression using Sangerbox. **p* < 0.05.


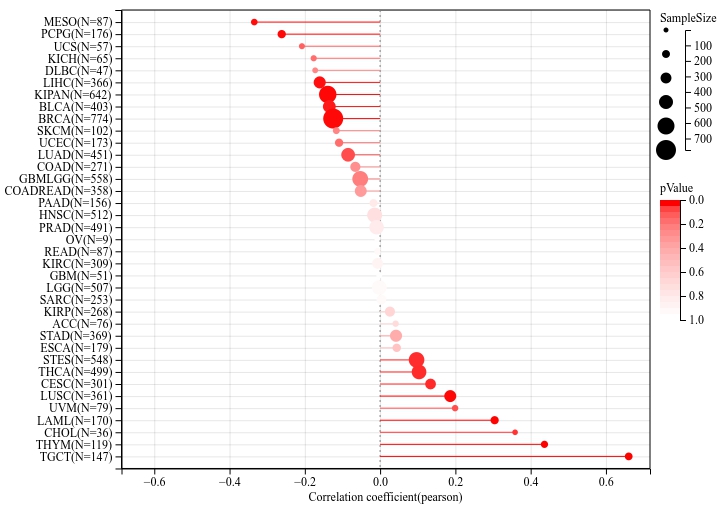


**Figure S12** Correlation between KLF4 expression and cancer stemness scores (DNAss) based on Pearson’s correlation analysis.
